# Bandage for the Cure of Prolapsus Uteri

**Published:** 1838

**Authors:** 


### Art. XIII.—Bandage for the cure of Prolapsus Uteri.

Dr. Robert Thompson, of Columbus, Ohio, has invented an apparatus for the cure or palliation of *prolapsus uteri*, which we have not had an opportunity of testing, but which seems well in appearance; and, in his own practice, we are told, has answered every desirable end. It makes firm pressure around the pelvis, holds up the abdominal viscera, and supports the perinœum and vulva. The Doctor exhibited it to the late Medical Convention, at Columbus, and made such explanatory remarks, concerning its *modus operandi* and effects, as seemed to convince that body of its superior utility. D.

